# Molecular characterisation of virulence genes in bacterial pathogens from daycare centres in Ile-Ife, Nigeria: implications for infection control

**DOI:** 10.1186/s12879-024-10095-8

**Published:** 2024-10-23

**Authors:** Eunice Damilola Wilkie, Jude Oluwapelumi Alao, Toyosi Teniola Sotala, Anthonia Olufunke Oluduro

**Affiliations:** 1 Microbiology Department, Adeleke University, Ede, Osun State Nigeria; 2 School of Public Health and Interdisciplinary Studies, Auckland University of Technology, Auckland, New Zealand; 3 Department of Microbiology, Obafemi Awolowo University, Ile-Ife, Osun State Nigeria

**Keywords:** Daycare centres, *Klebsiella pneumoniae*, *Staphylococcus aureus*, Virulence genes, Nigeria

## Abstract

**Background:**

Daycare centres play a critical role in early childhood development but are high-risk environments for infectious disease transmission due to close physical contact, shared toys, inadequate hygiene, and poor ventilation. These risks are especially concerning in low- and middle-income countries (LMICs) like Nigeria, where resources for infection control may be limited. This study aimed to identify and characterise virulence genes in bacterial isolates from daycare centres in Ile-Ife, Nigeria, to assess infection risks.

**Methods:**

Between November 2017 and July 2019, 233 samples were collected from 76 children, 33 daycare workers, and 124 fomites in 17 daycare centres. The bacterial isolates were analysed using conventional PCR and RAPD analysis to detect the presence of virulence genes. The frequency of crucial virulence genes and the prevalence of each bacterial species were recorded.

**Results:**

Key virulence genes were detected, including *fimH* in *Klebsiella species* (22.73% of Gram-negative isolates), *algD* in *Pseudomonas aeruginosa* (50%), and *icaA* and *cna* in *Staphylococcus aureus* (16.67%). *Staphylococcus aureus* was the most prevalent species (35%), followed by *Klebsiella* (28%) and *Pseudomonas aeruginosa* (20%).

**Conclusion:**

This study highlights the presence of virulent bacterial pathogens in daycare environments, posing a severe infection risk to children. To mitigate these risks, it is essential to implement enhanced infection control measures, such as regular microbial screening, improved hand hygiene practices, and disinfection protocols for fomites. Training programs for daycare workers on hygiene practices and routine monitoring could also significantly reduce infection transmission. These interventions are vital for safeguarding the health of daycare children in Nigeria and similar settings globally.

**Supplementary Information:**

The online version contains supplementary material available at 10.1186/s12879-024-10095-8.

## Introduction

Daycare centres are critical environments for the social and cognitive development of young children; however, they also present significant public health challenges due to the high susceptibility of children to infectious diseases. The close physical interactions among children, frequent sharing of toys, and inadequate disinfection of fomites create ideal conditions for the transmission of bacterial pathogens [1]. These pathogens often harbour virulence genes that enhance their ability to colonise, invade, and cause disease in hosts, making them particularly dangerous in such communal settings. Infections in children can lead to serious health outcomes, including diarrhoea, respiratory infections, and skin diseases, which contribute significantly to childhood morbidity and mortality. Globally, infections are one of the leading causes of death in children under five, with developing countries bearing the brunt of this burden.

In Nigeria, particularly in low- and middle-income countries (LMICs), several factors have heightened the risk of bacterial infections in daycare centres. These include overcrowding, limited access to clean water, poor sanitation, and a lack of regular disinfection protocols [2, 3]. A study on Nigerian daycare centres reported high contamination of toys and other fomites, contributing to frequent infections among attendees [4]. However, there is limited data on the specific prevalence of bacterial pathogens and the virulence genes they carry in these settings, particularly in South-Western Nigeria. Existing research from other African countries has highlighted similar issues, with bacterial pathogens such as *Staphylococcus aureus* and *Escherichia coli* commonly detected in children [5, 6]. The gap in data from Nigerian daycare centres necessitates further investigation into the types of bacterial pathogens present and their potential to cause severe health outcomes in children.

Daycare centres in Ile-Ife, Nigeria, typically operate under resource-limited conditions. These centres often struggle with overcrowding, poor ventilation, inadequate hand hygiene, and limited sanitation facilities, all of which facilitate the spread of infections. In such environments, children are at an increased risk of contracting bacterial infections that can lead to complications such as malnutrition, prolonged illness, hospitalisation, and even death. The public health burden of these infections is substantial, as they not only affect the well-being of children but also place significant strain on healthcare systems in Nigeria. Frequent infections in early childhood have long-term consequences, including impaired growth and development, which can affect children’s future health and educational outcomes [7].

This study addresses the lack of molecular data on bacterial pathogens in Nigerian daycare centres by identifying and characterising virulence genes in bacterial isolates from selected daycare facilities in Ile-Ife. Using polymerase chain reaction (PCR) and Random Amplified Polymorphic DNA (RAPD) analysis, the research focuses on determining the prevalence and distribution of crucial virulence genes such as *fimH, algD, icaA*, and *cna*. These “advanced molecular techniques” allow for precise detection and analysis of virulence factors, which contribute to the pathogenicity of the bacteria. By characterising these genes, we aim to provide crucial insights into the infection risks in daycare centres and offer evidence-based recommendations for infection control measures.

The relevance of this study extends beyond academic interest; it has important implications for public health policy and practice in Nigeria and similar LMICs. By understanding the virulence profiles of bacterial pathogens in daycare centres, health authorities can develop targeted interventions, such as regular microbial screening, improved hygiene protocols, and infection control training for daycare workers. These findings will inform local and national health policies aimed at reducing the burden of infectious diseases in communal settings, ultimately helping to create safer environments for young children.

## Methods

### Ethical considerations

This study received ethical approval from the Health Research Ethics Committee (HREC) at the Institute of Public Health, Obafemi Awolowo University, Ile-Ife, Nigeria (HREC Number: IPHOAU/12/1337). Written informed consent was obtained from the parents of children attending the daycare centres and the daycare workers before sample collection. Participant confidentiality was maintained by anonymising all data collected from the centres.

### Sample collection

A systematic sampling approach was employed to select daycare centres, ensuring representation from urban and rural areas in Ile-Ife. The study, conducted between November 2017 and July 2019, included 20 daycare centres were visited twice to collect samples. Samples were collected from fomites (toys, diaper changing areas, tables, mats, door handles, and bedsheets) and from the palms, fingers, and nostrils of children and daycare workers using sterile cotton swabs moistened with sterile saline. These fomites and body parts were chosen based on their frequent contact and documented association with bacterial transmission in similar environments [1]. They were transported to the laboratory within two hours in Amies transport medium (Thermo Fisher Scientific, Waltham, MA, USA).

### Questionnaire administration and data collection

Parents, guardians, and daycare workers were interviewed using a validated structured questionnaire (Supplementary file 1), which was pre-validated before the study by the HREC. The questionnaire gathered socio-demographic data, medical history of the children, and occupational information of the parents/guardians and workers. The validation process involved expert reviews to ensure the data collection tool was reliable for the study population.

### Inclusion and exclusion criteria

Inclusion Criteria:

- Children aged 6 to 42 months attending the selected daycare centres.
- Daycare workers directly involved in childcare.

Exclusion Criteria:

- Parents or guardians who refused consent.
- Children over 42 months of age.
- Children receiving antibiotic therapy at the time of the study, as antibiotics could interfere with bacterial isolation.

The chosen age range (6 to 42 months) was based on the critical developmental period when children have the highest attendance rates in daycare centres and increased exposure to potential pathogens.

### Isolation and identification

In the laboratory, swabs were vortexed in 1 mL of sterile phosphate-buffered saline (PBS) to release bacteria. The resulting suspension was cultured on various media, including Nutrient agar, Eosin Methylene Blue (EMB) agar, Muller Hinton agar, MacConkey agar, Triple Sugar Iron agar, Sulphide Indole Motility agar, Mannitol Salt agar, and Blood agar plates (Oxoid, Himedia). Plates were incubated at 37 °C for 24–48 h. Biochemical tests were conducted to identify bacterial isolates, such as catalase, citrate, indole, methyl red, Voges-Proskauer, motility, oxidase, hydrogen sulphide production, DNase, and spore formation tests. Gram-negative isolates were further identified using the Microbact™ GNB 24E identification kit.

### Antibiotic susceptibility testing

Antibiotic susceptibility was assessed using the Kirby-Bauer disk diffusion method on Mueller–Hinton agar plates, following Clinical and Laboratory Standards Institute (CLSI) 2020 guidelines [8]. The selection of antibiotics was based on common prescriptions and documented resistance patterns in Nigeria [9, 10]. For Gram-positives, antibiotics (Biomark Laboratories, India) used included gentamicin (10 µg), amoxicillin/clavulanic acid (30 µg), ceftazidime (30 µg), cephalexin (1.5 µg), cefuroxime (10 µg), erythromycin (5 µg), vancomycin (30 µg), cotrimoxazole (25 µg), ampicillin (10 µg), tetracycline (30 µg), ciprofloxacin (5 µg), cefuroxime (10 µg), and ceftazidime (10 µg). For Gram-negative bacteria, antibiotics (Abtek Biological Ltd and Oxoid, England) included gentamicin (10 µg), tetracycline (10 µg), meropenem (10 µg), cotrimoxazole (30 µg), chloramphenicol (30 µg), ciprofloxacin (5 µg), trimethoprim (5 µg), nitrofurantoin (300 µg), ofloxacin (5 µg), amoxicillin/clavulanic acid (30 µg), and cefotaxime (30 µg).

### Molecular analysis for virulence genes

#### DNA extraction

Bacterial DNA was extracted using a boiling method for its simplicity and effectiveness. Although more robust methods such as bead beating or enzymatic lysis are available, this method was chosen due to its cost-effectiveness and adequate yield for PCR analysis. The DNA extraction involved suspending bacterial colonies in 200 µL of sterile distilled water, boiling the suspension at 100 °C for 15 min, and centrifuging it at 10,000 rpm for 15 min. The supernatant containing the DNA was used for PCR analysis.

#### Polymerase Chain Reaction (PCR) for *Staphylococcus* aureus detection

Primers specific for the *Staphylococcus aureus* *STPY* gene were used (Table 1), as *S. aureus* is a common pathogen in community settings, often associated with skin infections and poor hygiene [11]. The PCR reaction mixture12.5 µL of master mix, 0.5 µL each of forward and reverse primers, 8.5 µL of nuclease-free water, and 3 µL of template DNA. PCR conditions involved initial denaturation at 94 °C for 5 min, followed by 37 cycles of denaturation at 94 °C for 1 min, annealing at 50 °C for 30 s, and extension at 72 °C for 1 min, with a final extension at 72 °C for 5 min. The thermocycler (Applied Biosystems, Singapore) was used for PCR amplification.

**Table 1:** Primers for the Detection of* S. aureus*

Gene	Primer Sequence (5’-3’)	Size (bp)	Reference
*STPY*	F: ACGGTCTTGCTGTCACTTATA	257	[12]
	R: TACACATATGTTCTTCCCTAATAA		

#### Gel electrophoresis

PCR products were separated on a 1.5% agarose gel prepared by dissolving 1 g of agarose powder (Cleaver Scientific, UK) in 100 mL of 1X Tris–Borate-EDTA (TBE) buffer (Bioconcept, Ltd, Switzerland). The solution was heated in a microwave for 2–3 min until clear, cooled to approximately 50 °C, and mixed with 5 µL of ethidium bromide. The mixture was poured into a tray with combs to form wells. After setting, the gel was placed in an electrophoresis tank with 1X TBE buffer. Each sample (5 µL amplicon mixed with 5 µL loading buffer) was loaded into the wells. Electrophoresis was performed at 100 V for 25 min. A 100 bp DNA ladder was used as a size marker [13]. DNA bands were visualised under a shortwave UV transilluminator and photographed using a gene gel bioimaging system. PCR products were analysed by comparing DNA bands with the 100 bp DNA standard.

#### Detection of virulence genes in gram-negative bacterial isolates

Multiple antibiotic-resistant isolates were selected based on their antibiotic susceptibility profiles. They were tested for virulence genes (*IroN, toxA, Biofilm*, *PapC, fimH,* and *algD*) using specific primers (Table 2). Each PCR mixture contained 12.5 µL of master mix, 0.5 µL each of forward and reverse primers, 8.5 µL of nuclease-free water, and 3 µL of template DNA. The PCR conditions for detecting *toxA, IroN,* and *Biofilm* genes were an initial denaturation at 94 °C for 5 min, 35 cycles of denaturation at 94 °C for 1 min, annealing at 47 °C for 1 min, extension at 72 °C for 2 min, and a final extension at 72 °C for 10 min. For detecting *PapC, fimH,* and *algD* genes, the annealing temperature was 42 °C.

**Table 2:** Primers used for the detection of virulence genes in selected gram-negative bacterial isolates

**Gene**	**Primer Sequence (5’-3’)**	**Size (bp)**	**References**
*PapC*	F: TGATATCACGCAGTCAGTAGC R: CCGGCCATATTCACATAA	501	[14]
*IroN*	F: AATCCGGCAAAGAGACGAACCGCCT R: GTTCGGGCAACCCCTGCTTTGACTTT	533	[14]
*Biofilm*	F: GATTCAATTTTGGCGATTCCTGC R: TAATGAAGTCATTCAGACTCATCC	225	[15]
*fimH*	F: TACTGCTGATGGGCTGGTC R: GCCGGAGAGGTAATACCCC	640	[15]
*algD*	F: CGTCTGCCGCGAGATCGGCT R: GACCTCGACGGTCTTGCGGA	313	[16]
*toxA*	F: GGTAACCAGCTCAGCCACAT R: TGATGTCCAGGTCATGCTTC	352	[17]

Key: F- Forward; R- Reverse; bp- base pairs

#### Virulence genes and primer validation

The study targeted several key virulence genes associated with bacterial pathogenicity. The *PapC* gene encodes a protein that is crucial for the assembly of *Pili* or *fimbriae*, which are hair-like structures on the surface of bacteria that facilitate adhesion to host epithelial cells, particularly in urinary tract infections [18]. The *IroN* gene encodes a receptor involved in iron acquisition, a critical process for bacterial survival in iron-limited environments such as the human body and contributes to the virulence of pathogens by enhancing their ability to thrive during infection [19].

The *Biofilm* gene is associated with the ability of bacteria to form biofilms, structured communities of bacteria that adhere to surfaces and are protected by an extracellular matrix. Biofilm formation is significant in chronic infections, enabling bacteria to resist both the host immune response and antibiotic treatment [20]. The *fimH* gene encodes a mannose-binding adhesin located at the tip of *type 1 fimbriae*, which plays a crucial role in bacterial attachment to host cells, particularly in the urinary tract, enhancing colonisation and persistence of infection [21].

The *algD* gene is involved in the biosynthesis of alginate, a polysaccharide that protects bacteria from host immune responses and is crucial for biofilm formation in respiratory infections [22]. The *toxA* gene encodes exotoxin A, a potent virulence factor inhibiting protein synthesis in host cells, leading to cell death and contributing to tissue damage in infections caused by certain bacterial species [23].

#### Detection of virulence genes in *S. aureus*

Selected multiple antibiotic-resistant *S. aureus* isolates were tested for *icaA, cna,* and *fnbA* genes using specific primers obtained from Inqaba Biotechnical Industries (Pty) Ltd (Table 3). For *icaA* and *cna*, each PCR vial contained master mix, forward and reverse primers, nuclease-free water, and 3 µL of template DNA. The PCR conditions were an initial denaturation at 94 °C for 5 min, followed by 37 cycles of denaturation at 94 °C for 1 min, annealing at 43 °C for 30 s, and extension at 72 °C for 2 min, with a final extension at 72 °C for 7 min. For *fnbA,* the annealing temperature was 46 °C.

**Table 3:** Primers used for the detection of virulence genes in selected *S. aureus*

Gene	Primer Sequence (5’-3’)	Size (bp)	References
*fnbA*	F: CATAAATTGGGAGCAGCATCA R: ATCAGCAGCTGAATTCCCATT	128	[24]
*cna*	F: AAAGCGTTGCCTAGTGGAGA R: AGTGCCTTCCCAAACCTTTT	192	[24]
*icaA*	F: GATTATGTAATGTGCTTGGA R: ACTACTGCTGCGTTAATAAT	770	[25]

Key: F- Forward; R- Reverse; bp- base pairs

### PCR for genetic relatedness using Random Amplified Polymorphic DNA (RAPD) technique

Randomly selected *K. pneumoniae* isolates were analysed using primers OP-A3, OP-A4, and OP-A7 (Table 4). Each PCR vial contained 12.5 µL of master mix, 1.0 µL of each primer, 8.5 µL of nuclease-free water, and 3 µL of template DNA. The PCR conditions included initial denaturation at 94 °C for 5 min, followed by 40 cycles of denaturation at 94 °C for 30 s, annealing at 29 °C for 1 min 5 s, extension at 72 °C for 2 min 5 s, and a final extension at 72 °C for 7 min.

**Table 4:** Primers used for the profiling of random amplified polymorphic dna in selected bacterial isolates

**Primer Name**	**Sequence**	**Reference**
*OP-A3*	AGTCAGCCAC	[26]
*OP-A4*	AATCGGGCTG	[26]
*OP-A7*	GAAACGGGTG	[26]

### Gel electrophoresis for RAPD-PCR

PCR products were separated on a 1.5% agarose gel using the same method as described previously. Each sample mixture (5 µL of amplicon mixed with 5 µL of loading buffer) was loaded into the wells. Electrophoresis was carried out at 100 V for 25 min. DNA bands were visualised and photographed using a UV transilluminator and a gene gel bioimaging system. PCR products were analysed by comparing DNA bands with a 100 bp DNA standard.

#### Assessment of genetic relatedness using RAPD-PCR

The genetic relatedness among various strains was assessed using RAPD-PCR with specific primers obtained from Inqaba Biotechnical Industries (Pty) Ltd [27]. Amplified profiles were compared, scoring DNA fragments as “1” for presence and “0” for absence to generate a binary matrix. Percent polymorphism was calculated, and pairwise genetic similarities were estimated using the DICE similarity coefficient. Clustering was performed using the unweighted pair group method with arithmetic mean (UPGMA) and analysed with DARWIN 5.0 software.

## Results

### Baseline characteristics of the study population

The study period was between November 2017 and July 2019. A total of 233 samples were collected from selected daycare centres within Ile-Ife, including 76 children, 33 daycare workers, and 124 fomites (Table 5). Out of 20 schools visited, 17 agreed to participate in the study.

**Table 5:** Baseline characteristics of the study participants

Characteristic	Cases n (%)
**Sex:**	
Male	40
Female	36
**Mother’s Occupation:**	
Public servants	41
Professionals/Managers	4
Farmers/traders/artisans	19
Students	3
Dependent/unemployed	0
Others	9
**Children’s Age Group (months):**	
6–12 months	17
13–24 months	40
25–36 months	17
36–42 months	2

### Cultural and morphological characteristics of *bacteria* isolated from fomites, hands, nostrils of children, and daycare workers

The cultural and morphological characteristics of the bacteria isolated from fomites, hands, and nostrils of children and daycare workers. *S. aureus* isolates were observed as yellowish and non-mucoid on Mannitol salt agar, while other *Staphylococcus* species were pinkish. *Escherichia coli* appeared large and pink-rose on MacConkey agar. *Bacillus* sp. were moderate to large, irregular in shape, and whitish on nutrient agar. *Pseudomonas aeruginosa* were moderate in size, entire in shape, raised, and greenish on nutrient agar. *Corynebacterium* species were brownish on nutrient agar, raised, and entire in shape.

### Molecular detection of virulence genes in *Enterobacteriaceae*

**Fig. 1:**
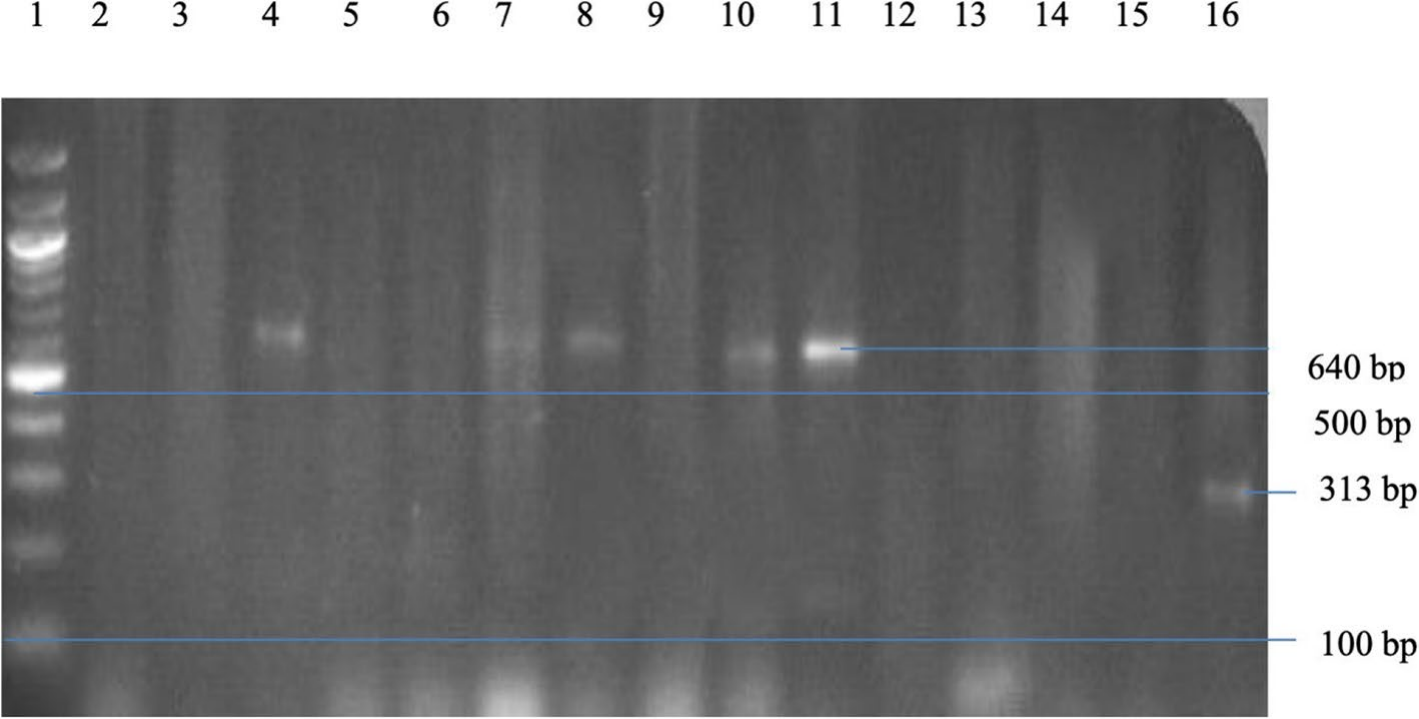
Agarose Gel Electrophoresis of Multiplex PCR Products for *fimH* (640 bp) and *algD* (313 bp) Genes in Selected Bacterial Isolates. Lane 1: 100 bp Ladder, Lane 4: *K. pneumoniae*, Lane 7: *Enterobacter agglomerans*, Lanes 8, 10, 11: *K. oxytoca*, Lane 16: *P. aeruginosa*

The molecular detection of virulence genes in *Enterobacteriaceae* revealed the *fimH* gene (640 bp) presence in 5 out of 22 representative Gram-negative bacterial isolates, accounting for 22.73%. Additionally, the *algD* gene (313 bp) was identified in 1 out of 2 *P. aeruginosa* isolates, representing 50%. The gel electrophoresis results are illustrated in Fig. 1, and the frequency and percentage distribution of virulence genes in Gram-negative bacteria are summarised in Table 6.

**Table 6:** Distribution and prevalence of virulence genes in gram-negative bacteria: frequency and percentage analysis

**Bacteria Isolate**	***fimH***		***algD***		***PapC***		***Biofilm***		***IroN***		***toxA***	
	Positive	Negative	Positive	Negative	Positive	Negative	Positive	Negative	Positive	Negative	Positive	Negative
*K. oxytoca*	3 (60%)	2 (40%)	NA	NA	0 (0%)	5 (100%)	0 (0%)	5 (100%)	0 (0%)	5 (100%)	NA	NA
*K. pneumoniae*	1 (33.3%)	2 (66.67%)	NA	NA	0 (0%)	3 (100%)	0 (0%)	3 (100%)	0 (0%)	3 (100%)	NA	NA
*E. coli*	0 (0%)	1 (100%)	NA	NA	0 (0%)	1 (100%)	0 (0%)	1 (100%)	0 (0%)	1 (100%)	NA	NA
*P. rettgeri*	0 (0%)	2 (100%)	NA	NA	0 (0%)	1 (100%)	0 (0%)	1 (100%)	0 (0%)	2 (100%)	NA	NA
*P. stuarti*	0 (0%)	1 (100%)	NA	NA	0 (0%)	1 (100%)	0 (0%)	1 (100%)	0 (0%)	1 (100%)	NA	NA
*E. agglomerans*	1 (20%)	4 (80%)	NA	NA	0 (0%)	5 (100%)	0 (0%)	5 (100%)	0 (0%)	5 (100%)	NA	NA
*C. youngae*	0 (0%)	1 (100%)	NA	NA	0 (0%)	1 (100%)	0 (0%)	1 (100%)	0 (0%)	1 (100%)	NA	NA
*E. clocae*	0 (0%)	1 (100%)	NA	NA	0 (0%)	1 (100%)	0 (0%)	1 (100%)	0 (0%)	1 (100%)	NA	NA
*P. mirabilis*	0 (0%)	2 (100%)	NA	NA	0 (0%)	2 (100%)	0 (0%)	2 (100%)	0 (0%)	2 (100%)	NA	NA
*P. aeruginosa*	NA	NA	1 (50%)	1 (50%)	0 (0%)	NA	NA	NA	NA	NA	0 (0%)	2 (100%)
*S. liquefaciens*	0 (0%)	1 (100%)	NA	NA	0 (0%)	1 (100%)	0 (0%)	1 (100%)	0 (0%)	1 (100%)	NA	NA

*NA* Not available

### Molecular detection of virulence genes in *S. aureus*

**Fig. 2:**
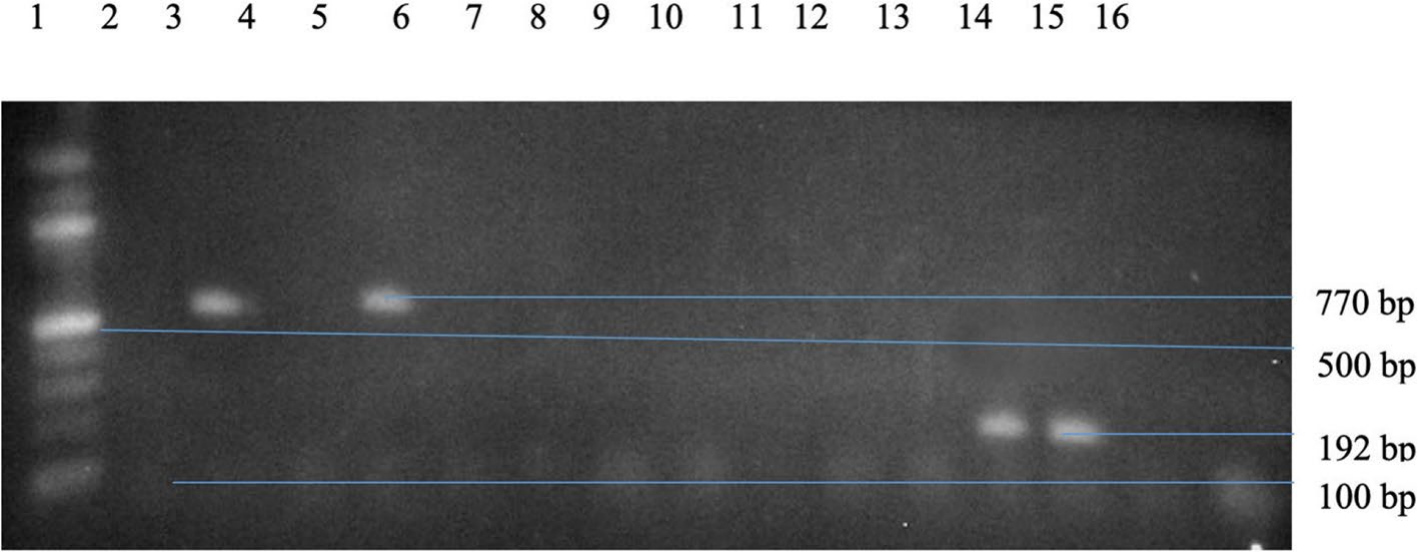
Agarose Gel Electrophoresis of Multiplex PCR Products for *icaA* (770 bp) and *cna* (192 bp) Virulence Genes in *S. aureus*. Lane 1: 100 bp Marker, Lanes 3 & 5: *icaA* Gene, Lanes 13 & 14: *cna* Gene

The molecular analysis of virulence genes in *S. aureus* indicated that 2 out of 13 isolates (16.67%) were positive for both the *icaA* (770 bp) and *cna* (192 bp) genes. The corresponding gel electrophoresis results are presented in Fig. 2. Table 7 shows the frequency and percentage distribution of virulence genes in *S. aureus.*

**Table 7:** Frequency and percentage distribution of virulence genes in *S. aureus* isolates

Virulence Gene	Positive Cases (*N* = 13)	Percentage (%)
*icaA*	2	16.67%
*cna*	2	16.67%
*fnbA*	0	0.00%

Key: *N* = Total number of *S. aureus* isolates

### RAPD pattern of *K.pneumoniae*

**Fig. 3:**
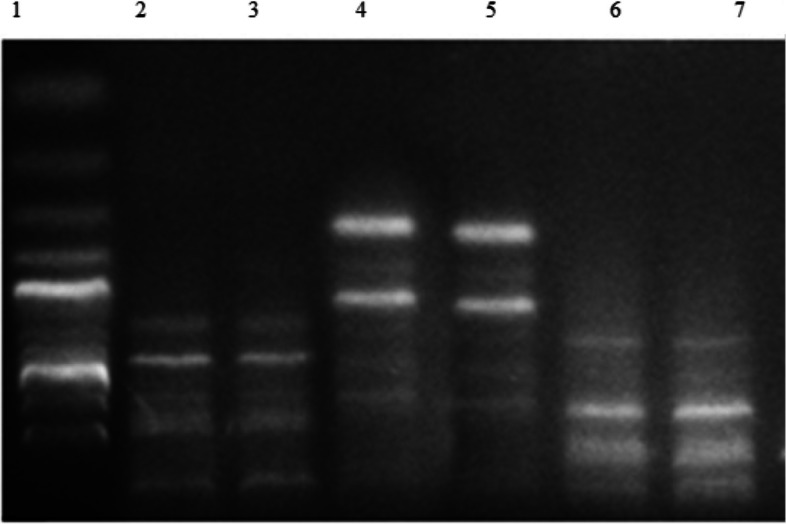
Gel Electrophoresis of RAPD Profiles for *K. pneumoniae* Isolates from Daycare Workers (Lanes 2–19) with Molecular Ladder (Lane 1)

**Fig. 4:**
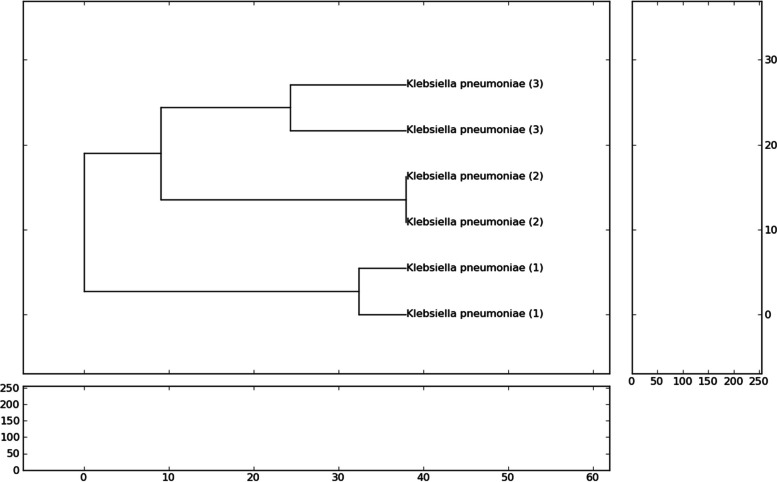
Dendrogram of *K. pneumoniae* Isolates from Nostril and Hand of Daycare Workers Based on RAPD Analysis

The RAPD analysis of* K. pneumoniae* isolates revealed a total of 28 bands, all polymorphic, ranging from 8 to 10 bands per isolate. Table 8 summarises the percentage similarity and genetic relatedness of the isolates in relation to multiple antibiotic resistance. The banding profiles generated by three different primers are shown in Fig. 3. The RAPD dendrogram, depicting the phylogenetic tree and highlighting the close genetic relationship between two specific isolates, is illustrated in Fig. 4.

**Table 8:** Polymorphic bands of each genetic primer and percentage of polymorphism in *K. pneumoniae*

Primer	Total Bands	Monomorphic Bands	Polymorphic Bands	Monomorphic Bands (%)	Polymorphic Bands (%)
OP-A3	10	0	10	0.00%	100%
OP-A4	10	0	10	0.00%	100%
OP-A7	8	0	8	0.00%	100%
Total	28	0	28	-	-

## Discussion

The findings of this study hold significant public health implications, particularly concerning infection control in daycare centres, where young children are highly vulnerable to infectious diseases. The detection of virulence genes in bacterial isolates from fomites, hands, and nostrils emphasises the potential for these environments to serve as reservoirs and transmission hubs for pathogenic bacteria.

The presence of the *fimH* gene in 22.73% of *Enterobacteriaceae* isolates, notably in *Klebsiella pneumoniae* and *K. oxytoca*, underscores the role of type 1 *fimbriae* in bacterial adhesion, invasion, and biofilm formation. These mechanisms are crucial for establishing infections in communal settings with frequent physical contact and shared surfaces, such as daycare centres. The lower prevalence of *fimH* compared to previous reports [15, 28], may reflect differences in bacterial species, sample sources, or regional factors. Nonetheless, the detection of this gene raises concerns about the potential for these bacteria to cause infections, reinforcing the need for stringent hygiene practices within such facilities.

The detection of the *algD* gene in 50% of *Pseudomonas aeruginosa* isolates aligns with its known role in producing alginate, a biofilm component that enhances bacterial persistence and resistance to antimicrobial agents. The absence of the *toxA* gene, which contrasts with findings from other regions [16, 29], suggests possible regional variations in the virulence profiles of *P. aeruginosa* strains. Given this bacterium’s role in both nosocomial and community-acquired infections, the presence of *algD* in daycare settings is alarming and underscores the need for targeted infection control measures. Strict cleaning, disinfection protocols, and reinforcing hand hygiene among staff and children are essential to prevent the spread of these resilient bacteria.

The absence of virulence genes like *PapC*, *Biofilm*, and *IroN* in *Enterobacteriaceae* isolates suggests these virulence factors may not be prevalent in the study population. However, the presence of other virulence genes, such as *fimH* and *algD*, remains a public health concern. These findings highlight the variability in virulence gene distribution and the complexity of bacterial pathogenicity. Ongoing surveillance is crucial to fully understand the risks posed by pathogenic bacteria in daycare environments and to develop appropriate interventions.

In *Staphylococcus aureus* isolates, the detection of *cna* and *icaA* genes in 16.67% of isolates is particularly concerning due to their roles in promoting bacterial adhesion and biofilm formation, both critical factors for establishing and maintaining infections [30, 31]. The identification of these genes in isolates from the nostrils of both children and daycare workers suggests potential for horizontal transfer within the daycare environment, further heightening the risk of infections. This is particularly troubling given *S. aureus’s* ability to cause a range of infections, including those affecting bones and joints [32]. The absence of the *fnbA* gene, despite its presence in other populations [33, 34], may reflect regional or environmental differences.

The RAPD analysis revealed significant genetic diversity among *K. pneumoniae* isolates, with 100% polymorphic bands, indicating high variability even within the same daycare centre. The genetic similarity observed between two isolates from different workers suggests possible transmission pathways within the daycare setting. The implication of this genetic diversity in terms of infection risk is profound, as diverse bacterial populations are more likely to evade immune responses and antimicrobial treatments, increasing the likelihood of persistent infections. This highlights the critical need for better infection control measures, including thorough cleaning and disinfection of surfaces, to prevent the spread of these genetically diverse and potentially resistant bacterial strains.

The study also suggests that daycare centres could serve as focal points for the dissemination of virulent and resistant bacterial strains, potentially spreading infections to households and the broader community. These findings have broader public health implications, emphasising the need for infection control guidelines specifically tailored for daycare settings. Public health policies should focus on establishing robust infection control standards and allocating resources for health interventions in such high-risk environments.

The molecular methods employed, particularly PCR, while valuable, could have been influenced by the limitations of sample contamination or sensitivity. Future studies should consider refining these methods and perhaps expanding to advanced techniques like whole-genome sequencing to provide a more comprehensive understanding of the molecular mechanisms underlying bacterial pathogenicity. Additionally, investigating a broader range of virulence genes and resistance markers would further illuminate the infection dynamics within daycare centres and inform more effective strategies for controlling infections in these vulnerable populations.

However, it is essential to consider the limitations related to the study’s geographical scope and sample size. The research was conducted in a specific region in Ile-Ife, Nigeria, which may not fully represent other regions or countries, and the relatively small sample size might affect the generalizability of the results. Future research should involve larger sample sizes and diverse geographic areas to capture a more comprehensive risk profile.

## Conclusion

This study highlights the significant public health risks posed by bacterial pathogens in daycare centres, particularly in resource-limited settings like Ile-Ife, Nigeria. The identification of key virulence genes, such as *fimH* in *Klebsiella* species, *algD* in *P. aeruginosa*, and *cna* and *icaA* in *S. aureus*, underscores the potential for these bacteria to cause serious infections. The findings emphasise the urgent need for improved infection control measures, including regular screening, enhanced hygiene practices, and targeted interventions to reduce the risk of infection in daycare centres. Addressing these challenges can better protect vulnerable children and prevent the spread of virulent and resistant bacterial strains. Limitations related to the study’s geographical scope and sample size may affect the generalizability of the findings, and future research should include larger sample sizes and advanced techniques like whole-genome sequencing to enhance understanding of bacterial pathogenicity. The insights gained from this study contribute to a broader understanding of bacterial pathogenicity in similar environments, paving the way for safer daycare settings globally.

## Supplementary Information

Supplementary Material 1.

## Data Availability

Data is provided within the manuscript or supplementary information files (Supplementary file 1).
