# Supplementary material for: Molecular characterisation of virulence genes in bacterial pathogens from daycare centres in Ile-Ife, Nigeria: implications for infection control
Source: BMC Infect Dis. 2024 Oct 23;24:1196. doi: 10.1186/s12879-024-10095-8 (PMC11515781; doi:10.1186/s12879-024-10095-8)
Supplement: Supplementary file 1 — Supplementary Material 1. [file 12879_2024_10095_MOESM1_ESM.docx]

**RESEARCH QUESTIONNAIRE**

**DEPARTMENT OF MICROBIOLOGY**

**FACULTY OF SCIENCE**

**OBAFEMI AWOLOWO UNIVERSITY, ILE-IFE, OSUN STATE**

**Topic** Phenotypic and Molecular Characterization of Bacterial Isolates in Fomites, Children and Workers in Some Day Care Centers in Ile-Ife, Nigeria

Dear Respondents,

Thank you for your willingness to participate, your child was selected by a scientific sampling procedure, and your cooperation is very important to the success of the study. This study will help provide information to parents and the workers handling the children on the need to improve the way the children are been cared for at home and at the Day care Centers  respectively since transfer of infection can be from hand to mouth, interaction with pets, fomites and many other means and children are known to be highly susceptible. This is a questionnaire you are asked to fill out on behalf of your child, please answer the questions as frankly and accurately as possible. ALL INFORMATION OBTAINED IN THE STUDY WILL BE KEPT CONFIDENTIAL AND USED FOR MEDICAL RESEARCH ONLY. You will be informed about the test results if you so desire.

**A.  SOCIO-DEMOGRAPHIC INFORMATION**

i. Name:                                          ii Sex:               iii Age:

iv. Father’s occupation………………..  v. mother’s occupation………………….

Father’s occupational level

Primary [ ]    secondary [ ]    university [ ]   others specify…………

Mother’s occupational level

Primary [ ]    secondary [ ]    university [ ]    others specify…….

Place of residence………………………………………………………………………….

**B. CLINICAL CONDITION**

1. History of previous or present infection YES [ ]         NO [ ] not sure [ ]

b. If YES, what is the nature of the infection? ………………………….

c. site of infection………………………….

d. Duration of the diagnosed infection ………………………………….

e. Has the infection been treated at any time? YES [ ] NO [ ] not sure [ ]

f. If YES, how was it treated? …………………………………………

g. has your child used antibiotics in the last 2 weeks ………………… YES [ ] NO [ ] not sure [ ]

h. if yes, what is the name of the antibiotics………………………………………………..

2. Do you have pets at home…………….YES [ ]   NO [ ]

3. What type of pet? Dog [ ]   Goat [ ] Cat [ ]    others specify……………………….

**C. DAY CARE WORKERS. (TO BE FILLED BY THE DAY CARE WORKERS ONLY)**

How often do you wash your hands…. Scarcely ------, once a day-------, frequently----

Do you use soap to wash your hands before handling the children YES [ ] NO [ ] not sure [ ]

Do you wash your hand after handling the children YES [ ] NO [ ] not sure [ ]

Do you disinfect your hand YES [ ] NO [ ] not sure [ ]

Do you have pets at home YES [ ] NO [ ]

What’s the name of the pet? Dog [ ]   Goat [ ] Cat [ ]    others specify……………………….

**CONSENT FORM**

I have read the letter of introduction to the study or it has been translated to me and I have had the opportunity to ask questions about the study

I understand that:

- My child’s nose and hands swab shall be collected by a trained medical personnel.
- My child’s medical information will remain confidential and will be used only for the purpose of the study.

I understand that I am free to refuse to take part in the study and if I choose to be part, I am free to leave at any time and without giving any reason, and that this will not affect my child.

I……………………………………………………………. hereby authorize that my child’s nose and hands swab should be collected and that my child’s medical information remains confidential.

Signature or thumbprint of the parent: …………………………………………..

Relationship: Father ( ) Mother ( ) Others (please specify) …………………………………….

Date: ………………………

Name of Child / Ward: ………………………………………..

Sex: ………………………………….. Age: …………………………

Researcher:  …………………………...                     ………………….   ………………

Name Sign Date
